# An Innovative Public–Private Mix Model for Improving Tuberculosis Care in Vietnam: How Well Are We Doing?

**DOI:** 10.3390/tropicalmed5010026

**Published:** 2020-02-14

**Authors:** Thuong Do Thu, Ajay M. V. Kumar, Gomathi Ramaswamy, Thurain Htun, Hoi Le Van, Luan Nguyen Quang Vo, Thuy Dong Thi Thu, Andrew Codlin, Rachel Forse, Jacob Crewsell, Hoi Nguyen Thanh, Hai Nguyen Viet, Huy Bui Van, Hoa Nguyen Binh, Nhung Nguyen Viet

**Affiliations:** 1 Vietnam Integrated Center for Tuberculosis and Respirology Research, Vietnam National Lung Hospital, Vietnam Tuberculosis Control Programme, Hanoi 100000, Vietnam; hoilv@yahoo.com (H.L.V.); nguyenviethai.hmu@gmail.com (H.N.V.); vhcapri@gmail.com (H.B.V.); nguyenbinhhoatb@yahoo.com (H.N.B.); VietNhung@yahoo.com (N.N.V.); 2 International Union Against Tuberculosis and Lung Disease, South-East Asia Office, New Delhi 110016, India; akumar@theunion.org; 3 International Union Against Tuberculosis and Lung Disease, Paris 75006, France; 4 Yenepoya Medical College, Yenepoya (Deemed to be University), Mangaluru 575018, India; 5 National Centre of Excellence and Advanced Research on Anemia Control, Centre for Community Medicine, All India Institute of Medical Sciences, New Delhi 110029, India; gmthramaswamy@gmail.com; 6 International Union Against Tuberculosis and Lung Disease, Mandalay 05021, Myanmar; thurainhtun30111990@gmail.com; 7 Friends for International Tuberculosis Relief, Ho Chi Minh City 700000, Vietnam; luan.vo@tbhelp.org (L.N.Q.V.); thuy.dong@tbhelp.org (T.D.T.T.); Andrew.codlin@tbhelp.org (A.C.); rachel.forse@tbhelp.org (R.F.); 8 Stop TB Partnership, Geneva 1218, Switzerland; jacobc@stoptb.org; 9 Haiphong International General Hospital, Haiphong 180000, Vietnam; hoinguyenthanhbm@gmail.com; 10 Hanoi Medical University, Hanoi 100000, Vietnam

**Keywords:** public–private mix model, public–private partnership, missing cases, operational research, SORT IT

## Abstract

To improve tuberculosis (TB) care among individuals attending a private tertiary care hospital in Vietnam, an innovative private sector engagement model was implemented from June to December 2018. This included: (i) Active facility-based screening of all adults for TB symptoms (and chest x-ray (CXR) for those with symptoms) by trained and incentivized providers, with on-site diagnostic testing or transport of sputum samples, (ii) a mobile application to reduce dropout in the care cascade and (iii) enhanced follow-up care by community health workers. We conducted a cohort study using project and routine surveillance data for evaluation. Among 52,078 attendees, 368 (0.7%) had symptoms suggestive of TB and abnormalities on CXR. Among them, 299 (81%) were tested and 103 (34.4%) were diagnosed with TB. In addition, 195 individuals with normal CXR were indicated for TB testing by attending clinicians, of whom, seven were diagnosed with TB. Of the 110 TB patients diagnosed, 104 (95%) were initiated on treatment and 97 (93%) had a successful treatment outcome. Given the success of this model, the National TB Programme is considering to scale it up nationwide after undertaking a detailed cost-effectiveness analysis.

## 1. Introduction

Tuberculosis (TB) is the leading cause of mortality from a single infectious agent globally, accounting for 1.45 million deaths annually [1]. The global community has pledged to end the TB epidemic by 2030 [2]. While there has been progress, the rate of decline of TB incidence has been modest at ~2% each year [3]. At this rate, we will not be able to realize the goal of ending TB by 2030. To accelerate progress, the Stop TB Partnership recommends that countries should strive to achieve 90-(90)-90 targets (diagnosing 90% of all people with TB including 90% among key populations and treating 90% of them successfully) [4].

One of the major challenges in TB control is “missing cases”. Globally, of the 10 million people estimated to have developed TB in 2018, only 7 million were notified [1]. The gap of 3 million includes people who are not diagnosed and treated, and those managed in the private health sector, but not notified to National Tuberculosis Programmes (NTP).

A multi-country study found that in more than 60% of TB patients, the private sector was the first point of contact, yet the proportion of cases notified to NTP was less than 10% [5]. Also, health care provision in the private sector is not standardized and poorly regulated in many countries. These may lead to delays in diagnosis and treatment, improper case management, increased risk of developing drug resistance, disease transmission, and catastrophic health expenditure [6]. Engagement and collaboration with private sector in those countries where a large proportion of care seeking is sought with private providers in critical [2].

Vietnam is one among the 30 high TB burden countries and has strong private health care sector. It is estimated that approximately 50% of TB patients seek initial care in the private sector before visiting the public health system [7,8]. In 2018, only 57% of estimated incident cases were notified to NTP, meaning there is no information about the rest of the patients and a majority of these may be receiving care in the private sector [1]. Mirroring the global picture, previous studies from Vietnam have also reported mismanagement in diagnosis and treatment of TB in the private health sector [9,10,11]. All these findings underline the need to engage the private health care providers in Vietnam’s TB care and prevention efforts. 

To engage the private sector, several public–private mix (PPM) models have been implemented by the Vietnam NTP since 2001. One such model involved training of private health care providers and strengthening referral mechanisms between the private sector and NTP. This yielded an increase in overall TB case detection rate of Ho Chi Minh City by 7% [12], but there were several gaps. About 30% of presumptive TB patients referred to NTP for sputum microscopy did not reach the diagnostic facility and nearly 60% of the patients diagnosed were lost to follow-up before starting treatment. Of those started on treatment, only 60% successfully completed it [13].

To address these gaps, a new model was implemented in Haiphong International General Hospital (HIGH) in 2018 as part of the TB REACH-funded Zero TB Vietnam initiative. This model included three unique components: (i) active facility-based screening of all adults for TB symptoms (and chest x-ray (CXR) for those with symptoms) by trained and incentivized providers, with on-site diagnostic testing or transport of sputum samples, (ii) an innovative mobile application to reduce dropout in the care cascade and iii) enhanced follow-up care through engagement of a local network of community health workers (CHWs). However, this model has not yet been systematically evaluated. In this study, we aimed to evaluate the performance of this private sector engagement model by tracking the cascade of tuberculosis care among the individuals attending the HIGH in Vietnam from June to December 2018.The specific objectives were to determine, (i) the number (proportion) with presumptive tuberculosis and among them, the number (proportion) who were investigated for tuberculosis (ii) the number (proportion) diagnosed with tuberculosis and initiated on treatment (iii) the treatment outcomes among those initiated on treatment and (iv) the delays involved at different steps of the care cascade.

## 2. Materials and Methods

### 2.1. Study Design

This was a cohort study involving analysis of routine surveillance data. 

### 2.2. Setting

Vietnam is a South-East Asian country with a population of 93.7 million. The country is divided into 63 provinces, which are further divided into districts, communes, and sub-communes. About 34.4% of the population live in urban areas and about 10% people are below the poverty line [14]. Healthcare services are provided by both public and private sectors.

#### 2.2.1. TB Control Program in Vietnam

The public health facilities for TB care and prevention are managed either directly by the NTP or indirectly through the Department of Health (DOH). Under the NTP, TB diagnosis, treatment, and control activities are carried out by the national level unit, 63 TB and lung disease hospitals or provincial TB units, and 707 TB management units (TBMUs) at the district level.

#### 2.2.2. PPM Models

There has been significant progress in the implementation of PPM initiatives for TB care and prevention in Vietnam. There are four types of PPM models. PPM Model 1 entails the referral of presumptive TB patients identified by the private providers to NTP for further evaluation. PPM Model 2 encompasses the diagnosis of TB and referral to the NTP. PPM Model 3 entails the provision of directly-observed treatment. PPM model 4 provides both diagnostic and treatment services similar to a TBMU. Private providers participating in PPM initiatives are trained for TB screening, diagnosis with sputum smear microscopy, and recording and reporting as per NTP guidelines.

#### 2.2.3. PPM Model at HIGH

PPM model at HIGH is similar to the model 4. HIGH was chosen for the intervention because it is the biggest tertiary care private hospital in Haiphong province (considered a poor-performing province by NTP) and there was political commitment from the director of the HIGH to collaborate with the NTP. Three departments from the HIGH (Endocrinology, Otolaryngology, and Respiratory medicine) were targeted for systematic screening. These three departments were chosen because they were expected to account for the majority of pulmonary presumptive TB patients visiting HIGH. The doctors and nurses of these departments were trained on the management of TB cases as per NTP guidelines and were given incentives for TB screening, diagnosis and treatment, and systematic recording and reporting. 

A total of 110 USD of fixed allowance per month was provided to the hospital. About 70 USD was provided every month for conducting the monthly review meetings and printing of forms. In addition, performance-based incentives were provided: (i) 0.5 USD given to the nurse for each chest X-ray conducted (ii) 1 USD given to the nurse for each sample transported for Xpert MTB/RIF testing (iii) 2 USD given to the doctor for each patient diagnosed with TB (iv) 3 USD given to the doctor for each TB patient completing treatment. The project was supervised by a focal point (@45 USD per month), PPM coordinator (@130 USD per month) and a PPM supervisor (@130 USD per month). These incentives were on top of the salaries they received. Thus, the total cost incurred on the project for six months was 4100 USD. All costs were incurred in Viet Nam Dong and translated to USD based on the average exchange rate during the implementation period.

All out-patients attending the selected departments (Endocrinology, Otolaryngology, and Respiratory medicine) were screened for symptoms such as cough, hemoptysis, chest pain and dyspnea, fever, fatigue and unexpected weight loss. Individuals who had any of these symptoms or who had a history of contact with TB were requested to undergo chest radiography. Contacts were defined as people living in the same household with a TB patient for at least two nights per week during the last 6 months. Individuals with symptoms suggestive of TB or exposure to a person with TB and parenchymal abnormalities suggestive of TB on chest X-ray were considered ‘presumptive TB’ and evaluated further for bacteriologic confirmation. The chest radiographs of the presumptive TB patients were examined by the radiologists at HIGH. Patients with TB symptoms and no abnormalities on chest X-ray were also offered tests for bacteriological confirmation based on the discretion of the attending physician. Spot sputum samples (without induction) were collected from the patients. For those who were not able to produce sputum, bronchoscopy was suggested by the treating physician and the bronchial washings were used for further testing. Patients with lymphadenopathy were referred for histopathological examination for confirmation of TB.

Individuals with a positive symptom screen were counselled by the treating physician about the different diagnostic tests available and their costs and were offered one of more of the following tests in the hospital for bacteriological confirmation: (i) Transcription Concerted Reaction (TRC) Ready 80 test (ii) sputum microscopy (iii) liquid culture and drug susceptibility test for first-line drugs. The TRC Ready 80 test is an automated molecular assay designed to detect mycobacterium tuberculosis (MTB) complex 16S rRNA present in clinical specimens (pulmonary and extra-pulmonary) or culture isolates. This has been described in greater detail elsewhere [15]. Though this test not endorsed by the WHO yet, it has been approved for use in Vietnam by the MOH for diagnosing TB. Chest X-ray screening and diagnostic tests at HIGH were paid by the patient at the following rates: 3 USD for chest X-ray, 3 USD for microscopy, 12 USD for culture, and 36 USD for TRC Ready 80. For patients unable to afford these tests, sputum samples were collected and transported to a nearby NTP facility for TB diagnosis using Xpert MTB/RIF assay, which was offered free of charge. The nurses in HIGH were trained on the procedures of sputum collection in falcon tubes, packaging and transportation in cold chain to the nearby NTP facility (which is located at a distance of 1.5 kms from HIGH). Nurses transported the sputum specimens in-person at the end of the day to the NTP facility. 

Patients diagnosed with TB disease either through bacteriologic confirmation or clinical diagnosis were initiated on anti-TB treatment at an NTP facility or a private health facility of the patient’s choice. Patients diagnosed with drug-susceptible TB were treated with first-line drugs and those with rifampicin resistance were referred to the NTP’s provincial TB hospital for further evaluation. While drugs were provided free of charge to the patients at NTP facilities, patients paid out of pocket at private health facilities. 

The CHWs were notified immediately after the diagnosis of each TB patient for linkage to care and follow-up from treatment start until completion. CHWs were motivated volunteers identified from each commune and recruited to support TB care and prevention activities in the Zero TB project (under funding support of TB REACH grant). They were mostly women and received formal training (for two days by NTP staff) on screening, counselling, follow-up care and support of TB patients. In the community, CHWs performed household contact tracing and counseling on treatment adherence and infection control. They received performance-based incentives as part of another project and did not receive any specific incentives for the project described in this study. The study’s case definitions and treatment outcomes were in accordance with NTP and WHO guidelines. 

#### 2.2.4. Recording and Reporting

All patient details were entered in the ACIS (Access to Care Information System, Clinton Health Access Initiative/TechUp, Vietnam) application, a data collection and case management tool for persons with presumptive TB in the community. Dedicated tablets with preinstalled software were procured and provided to the nurses and were trained on its use. Nurses captured data about presumptive TB patients using this tool. This application is bi-directionally connected with the NTP’s electronic recording and reporting system, the Vietnam TB Information Management Electronic System (VITIMES), which enabled the electronic referral of case files of persons with suspected or diagnosed TB to NTP facilities. 

### 2.3. Study Population

All patients aged ≥15 years attending the three outpatient departments of HIGH between 24 June, 2018 and 31 December, 2018 were included. 

### 2.4. Data Variables and Sources

Case-level covariates were extracted from the ACIS and VITIMES systems. These included TB symptoms, chest X-ray findings, diagnostic test results, diagnoses, treatment initiation dates, and treatment outcomes. We used name, age, and sex to merge the two databases, but removed names and other personal identifiers before analysis to ensure confidentiality of data.

### 2.5. Analysis and Statistics

Data was analyzed using Stata software (version 14.0, Statacorp, Texas, TX, USA). We have depicted the cascade of care in the form of a flowchart with dropouts at every stage summarized as frequencies and percentages. To calculate the proportion with presumptive TB, we chose all people attending the three out patient departments (OPD) as the denominator, because information on who among them were screened was not available in the records. TB treatment outcomes were categorized into successful (cured and treatment completed) and unsuccessful (failure, lost to follow-up, died, not evaluated) outcomes. The time delays between screening, undergoing diagnostic test and initiation of treatment were summarized using median and interquartile range (IQR). Factors associated with ‘not getting investigated for TB’ among presumptive TB patients were assessed using adjusted risk ratios and 95% confidence intervals (CI) calculated using log-binomial regression. We also assessed factors associated with TB diagnosis among patients investigated for TB using the same effect measures.

### 2.6. Ethics Approval

Ethics approval was obtained from the Scientific Ethics Committee of the National Lung Hospital, Hanoi, Vietnam (approval number 954/QD-BVPTU) and the Ethics Advisory Group of the International Union Against Tuberculosis and Lung Disease, Paris, France (approval number 22/19). Since this was a review of existing records with no direct interaction with human participants, the need for individual informed consent was waived by the ethics committees. Confidentiality of the patient data was ensured by (i) providing restricted access to patient data only to the research team (ii) using password protection to access the electronic files and (iii) removing all the personal identifiers (such as name, address, phone number) before analysis.

## 3. Results

A total of 52,078 adults attended the three OPDs at HIGH during the study period. Of them, 2739 (5.3%) had either symptoms suggestive of TB or were exposed to someone with TB. Of these, 1372 (50%) were male and mean (SD) age was 49 (17) years. The profile of these patients is depicted in Table 1. Cough was the predominant symptom present in 2240 (82%) individuals, followed by chest pain and dyspnea in 1926 (70%), fatigue in 1521 (56%) and fever in 418 (15%). Contact with a person with TB was reported by 34 (1%) patients.

### 3.1. Cascade of Care

**Figure 1:**
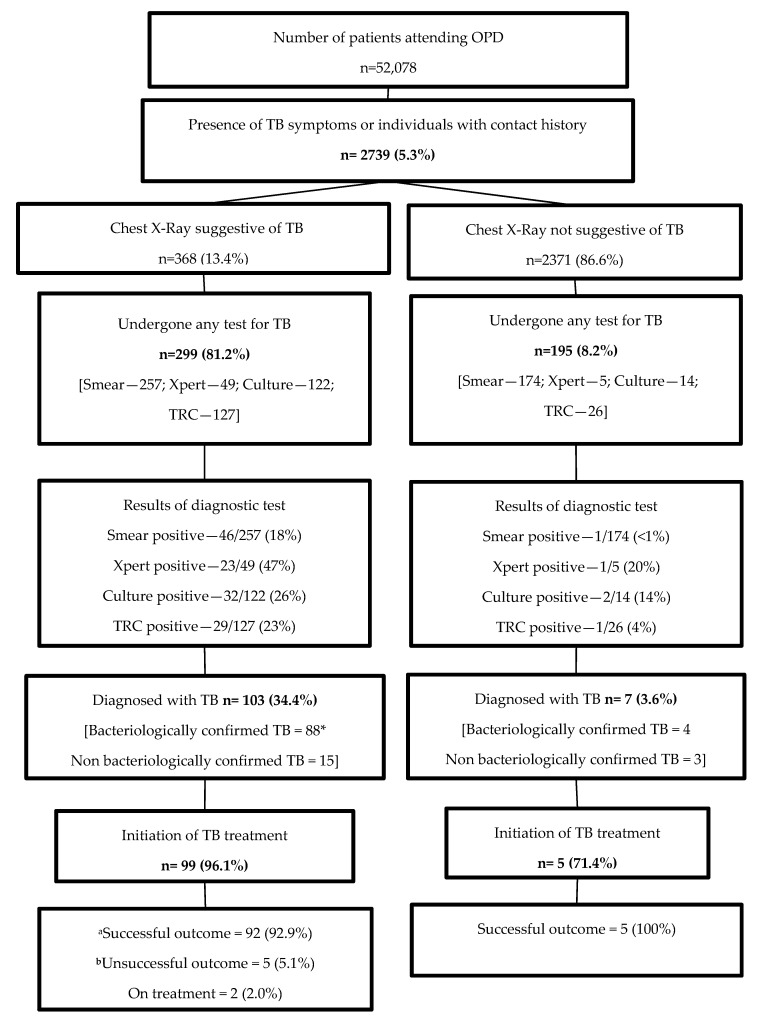
Cascade of tuberculosis care (from screening to treatment outcome) among the patients attending the Haiphong International General Hospital in Vietnam, from June to December 2018. OPD = Outpatient Department; TB = tuberculosis; TRC = transcription concerted reaction. a: successful outcome: cured and treatment completed. B: unsuccessful outcome: death, loss to follow-up, failure, and not evaluated. *Two individuals had rifampicin resistance and were started on second-line drugs.

The cascade of care among study participants is depicted in Figure 1. Of the 2739 symptomatic individuals or those with contact history, 368 (13.4%) had chest X-ray suggestive of TB and were identified as having presumptive TB (i.e., 368/52,078 [0.7%]). Of these, 299 (81%) underwent at least one of the diagnostic tests for TB and 103 (34.4%) were diagnosed with TB. In addition, 195/2371 (8%) patients with normal chest X-ray also underwent tests for TB, of whom 7 (4%) cases were diagnosed. In total, 110 people were diagnosed with TB. Of the 110 TB patients, 92 (84%) had bacteriologically confirmed TB and the rest were either clinically diagnosed or based on the results of other histopathological investigations. Of them, 104 (95%) were initiated on anti-TB treatment. All, except one, received treatment at an NTP health facility. Two individuals had rifampicin-resistant TB and were referred to the Provincial Lung Hospital and subsequently treated with second-line drugs. The majority of patients had pulmonary TB (n = 98, 88%), while the remaining 12 patients had extra-pulmonary TB (09 pleural TB and 3 lymph node TB).

### 3.2. Factors Associated with ‘Not Getting Tested for TB’

Of the 368 individuals with presumptive TB, 69 (19%) did not undergo any diagnostic testing. Patients without cough, without fever, without night sweats, without weight loss, and without TB contact history were less likely to be tested for TB (Table 2). 

### 3.3. Factors Associated with TB Diagnosis

Of the 494 patients who underwent diagnostic tests, 110 (22%) were diagnosed with TB. Patients with self-reported diabetes and those with weight loss had a significantly higher chance of getting diagnosed with TB (Table 3). 

### 3.4. Treatment Outcomes

The TB treatment outcomes are shown in Table 4. Among 104 patients initiated on treatment, 97 (93%) had successful treatment outcome, while 5 (5%) had unsuccessful outcome and 2 (2%) were still on treatment. 

### 3.5. Median Delays

The delays at different steps of the cascade are shown in Table 5. The median (IQR) duration from visiting the HIGH to undergoing TB diagnostic test was 0 (0–1) day and from diagnosis to initiation of treatment was 6 (1–17) days.

## 4. Discussion

This is the first report from Vietnam evaluating an innovative PPM model using an information technology based tool for improving tuberculosis care in private health sector. While there are many studies evaluating the specific components of the TB care cascade in the private sector, very few have comprehensively examined all the steps of the cascade in a single study [16]. This is one such effort. Overall, the performance of the model was excellent in plugging the gaps in TB care cascade in the private sector and substantially better than previous PPM models implemented in Vietnam [13]. About 80% of the presumptive TB patients were investigated for TB. Nearly 95% of the cases diagnosed were initiated on treatment, which is significantly better than previous studies from Vietnam and Pakistan, where nearly 60% were lost to follow-up before treatment [13,17]. All the cases were notified to NTP. More than 90% of all TB patients completed the treatment successfully, in line with the global 90-(90)-90 targets. These results were better than those reported from India [18], Pakistan [17], Thailand [19], and Vietnam [9,13] and were on par with outcomes reported from Myanmar [20].

In our view, the success of the model may be attributed to the following aspects. First, unlike earlier PPM initiatives which predominantly used a ‘referral model’ for investigation of tuberculosis (wherein presumptive TB patients were referred to an NTP facility), the new model offered TB tests on-site or arranged for transportation of sputum samples. This might have reduced the gaps and delays in testing. Second, the use of a mobile application enabled notification of every TB case diagnosed. This alerted the health care system and the last-mile service providers like CHWs to proactively track and provide follow-up care to the patients, thus reducing gaps in treatment initiation and completion. Third, all the providers were trained and performance-based incentives were offered for every successful event in the care cascade. The total costs incurred were modest at 4100 USD for the six-month pilot period (equivalent to ~37 USD per TB case diagnosed). However, we have not undertaken a detailed cost-effectiveness analysis. This should be a topic of future research. 

There were some other notable findings. First, only 0.7% of patients attending the OPD were identified as ‘presumptive TB’ patients. This is substantially lower than that reported from other settings like Pakistan (which varied from 2.9% to 7.5%) [21,22]. This difference is likely due to many differences between the settings which include (i) a stricter definition used for ‘presumptive TB’ (both symptom positive and chest X-ray abnormality) and (ii) the denominator being all patients attending OPD rather than the number screened in our study. It is possible that some of the patients attending the OPD might not have been screened and there was no documentation to find out the exact numbers screened. 

Second, about one in five presumptive TB patients did not undergo investigations and people without symptoms were less likely to undergo investigation. This is concordant with the observations by Creswell et al. in Karachi, Pakistan [22]. Patients without symptoms may have low risk perception or may have been accorded lower priority for testing by attending clinicians. Some patients may not have been able to produce a sputum sample. The high costs of the diagnostic tests for which patients had to pay out of pocket may have been another deterrent for uptake of tests. Also, people with symptoms but normal chest X-ray were less likely to be tested. This may be again related to the definition of presumptive TB used in this project, which required an abnormal X-ray in addition to symptoms. This may also be the reason for the high yield of TB (33%) among people with presumptive TB. 

Had we tested everyone with symptoms, we might have had lower yield in terms of percentage, but more cases in terms of absolute numbers. Of course, this would have had additional cost implications. One possible way to increase the number of cases detected without too much additional effort will be to use the duration of symptoms to prioritize investigation for TB—like investigating only those with cough of more than or equal to 2 weeks rather than testing everyone with cough of any duration [23]. Unfortunately, we did not have information on duration of symptoms and hence we cannot comment on this issue any further. All these call for revisiting the definition of presumptive TB used in the project.

Third, a standard diagnostic algorithm was not followed in the project. The nature and the number of tests offered to each patient seemed to vary. While we do not know the exact reasons for this variation, we speculate that this was dependent on the ability of individual patients to afford the high costs of the tests and based on the physician’s choice of diagnostic test. This aspect needs to be studied further using qualitative research methods. We recommend that all patients undergo a standard diagnostic algorithm, preferably using tests approved globally for use and, if possible, at subsidized costs.

The study had some limitations. First, we relied on routinely collected data and hence errors in documentation cannot be ruled out. However, we estimate that such errors are limited in number and impact given real-time and post hoc data validation mechanisms in the ACIS software and by data management team. Second, there was no documentation about the number of people screened. As a result, we were unable to calculate the ‘number needed to screen’ to detect an additional TB case. Also, the patients attending only three OPDs were screened. Hence the number of TB cases diagnosed may not reflect the true burden of TB among patients attending HIGH. We may have missed many patients, especially those with extrapulmonary TB because departments such as surgery, gynecology and urology were not involved in the project. We may also have missed many patients because of the strict definition of presumptive TB used in our study. In a national TB prevalence survey from Vietnam, only 10% of all presumptive TB patients fulfilled such strict criteria and accounted for only 27% of all TB patients [24]. Third, the study was conducted in a single hospital thereby limiting its generalizability. For this reason, we have refrained from the assessing the impact of this intervention on case notification at the community level. This kind of impact has been demonstrated by previous studies elsewhere [18,21,25]. Fourth, we did not have data for the pre-intervention period to enable before–after comparisons. There was no systematic recording and reporting of TB-related indicators before the study. The data obtained in this study may act as a baseline for any future evaluations. Finally, the exact reasons for the gaps at each step of the cascade were not investigated in this study. Future research should look into this aspect using qualitative research methods. 

In conclusion, the new PPM model in Vietnam performed well with high levels of testing, diagnosis, treatment start and completion among TB patients. Given the success of this model in plugging the gaps in TB care cascade, the NTP in Vietnam is considering to scale-up this model nationwide after undertaking a detailed cost-effectiveness analysis. The lessons learned from this study may be useful to make amendments in the PPM model and optimize project implementation going forward.

## Figures and Tables

**Table 1:** Socio-demographic and clinical profile of people with tuberculosis (TB) symptoms or contact history attending the Haiphong International General Hospital in Vietnam, from June to December 2018.

Characteristic	Number	(%)
**Total**	**2739**	**(100.0)**
**Age group (years)**		
15-44	1170	(42.7)
45-64	1084	(39.6)
≥ 65	485	(17.7)
**Gender**		
Male	1372	(50.1)
Female	1367	(49.9)
**Self-reported HIV status**		
Negative	596	(21.8)
Positive	4	(0.1)
Unknown	2139	(78.1)
**Self-reported diabetes**		
No	1699	(62.0)
Yes	161	(5.9)
Unknown	879	(32.1)
**Presenting symptoms**		
Cough (any duration) *	2240	(81.8)
Chest pain and dyspnea	1926	(70.3)
Fever	418	(15.3)
Fatigue	1521	(55.5)
Sweating at night	51	(1.9)
Unexplained weight loss	133	(4.9)
**History of contact of TB**	34	(1.2)

HIV = Human immunodeficiency virus infection, TB = Tuberculosis; * 125 of these patients had hemoptysis.

**Table 2:** Factors associated with ‘not getting tested for TB’ among presumptive TB patients attending the Haiphong International General Hospital in Vietnam, from June to December 2018.

Characteristic	Total	Not Tested for TB^#^		RR	(95%CI)	aRR	(95%CI)
		n	(%)				
**Total**	**368**	**69**	**(18.7)**	-		-	
**Age (years)**							
15–44	138	27	(19.6)	0.89	(0.52–1.51)	1.06	(0.97–1.08)
45–64	148	24	(16.2)	0.74	(0.43–1.28)	1.01	(0.96–1.07)
65 and above	82	18	(21.9)	1		1	
**Gender**							
Male	203	30	(14.8)	1		1	
Female	165	39	(23.6)	1.60	(1.04–2.46)	1.02	(0.99–1.06)
**Self-reported HIV status**							
Negative	195	24	(12.3)	-		-	
Positive	1	0	(0.0)	-		-	
Unknown	172	45	(25.2)	-		-	
**Self-reported diabetes**							
No	280	49	(17.5)	0.72	(0.39–1.34)	1.03	(0.94–1.13)
Yes	37	9	(24.3)	1		1	
Unknown	51	11	(21.6)	0.89	(0.41–1.92)	1.17	(1.06–1.28)
**Cough**							
No	68	20	(29.4)	1.80	(1.15–2.82)	1.07	(1.02–1.28)
Present	300	49	(16.3)	1		1	
**Fever**							
No	267	59	(22.1)	2.23	(1.19–4.19)	1.20	(1.12–1.29)
Present	101	10	(9.9)	1		1	
**Chest pain and dyspnea**							
No	128	23	(18.0)	0.94	(0.60–1.47)	0.99	(0.96–1.04)
Present	240	46	(19.2)	1		1	
**Fatigue**							
No	159	38	(23.9)	1.61	(1.05–2.47)	0.95	(0.91–0.98)
Present	209	31	(14.8)	1		1	
**Night sweat**							
No	343	68	(19.8)	4.96	(0.72–34.21)	1.37	(1.08–1.77)
Present	25	1	(4.0)	1		1	
Weight loss *							
No	311	65	(20.9)	2.93	(1.11–7.71)	1.38	(1.20–1.62)
Present	56	4	(7.1)	1		1	
**Contact history of TB**							
No	355	67	(18.8)	1.23	(0.34–4.47)	1.31	(1.01–1.71)
Present	13	2	(15.4)	1		1	

* Data missing for one; HIV = human immunodeficiency virus; TB = tuberculosis; CI = confidence interval; aRR = adjusted relative risk.

**Table 3:** Factors associated with diagnosis of TB among the patients who were investigated in the Haiphong International General Hospital in Vietnam, from June to December 2018.

Characteristic	Number Tested	Confirmed TB^#^		RR	(95%CI)	aRR	(95%CI)
		n	(%)				
**Total**	**494**	**110**	**(22.3)**	-		-	
**Age (years)**							
15–44	191	45	(23.6)	1.32	(0.81–2.16)	1.59	(0.93–2.72)
45–64	202	47	(23.3)	1.31	(0.80–2.13)	1.33	(0.80–2.21)
65 and above	101	18	(17.8)	1		1	
**Gender**							
Male	267	63	(23.6)	1.14	(0.82–1.59)	1.01	(0.73–1.39)
Female	227	47	(20.7)	1		1	
**Self-reported HIV status**							
Negative	218	70	(32.1)	-		-	
Positive	2	0	(0.0)	-		-	
Unknown	274	40	(14.6)	-		-	
**Self-reported diabetes**							
No	375	83	(22.1)	1		1	
Yes	40	16	(40.0)	1.81	(1.18–2.76)	2.13	(1.31–3.48)
Unknown	79	11	(13.9)	0.63	(0.35–1.12)	0.78	(0.44–1.39)
**Cough**							
No	66	10	(15.1)	1		1	
Present	428	100	(23.4)	1.54	(0.85–2.80)	1.42	(0.82–2.46)
**Fever**							
No	355	69	(19.4)	1		1	
Present	139	41	(29.5)	1.52	(1.08–2.13)	1.32	(0.95–1.83)
**Chest pain and dyspnea**							
No	162	44	(27.2)	1		1	
Present	332	66	(19.9)	0.73	(0.53–1.02)	0.78	(0.56–1.08)
**Fatigue**							
No	224	40	(17.9)	1		1	
Present	270	70	(25.9)	1.45	(1.03–2.05)	1.18	(0.82–1.69)
**Night sweat**							
No	469	100	(21.3)	1		1	
Present	25	10	(40.0)	1.88	(1.12–3.13)	1.31	(0.76–2.25)
Weight loss *							
No	433	83	(19.2)	1		1	
Present	60	27	(45.0)	2.35	(1.67–3.30)	2.06	(1.44–2.96)
**Contact history of TB**							
No	480	105	(21.9)	1		1	
Present	14	5	(35.7)	1.63	(0.79–3.36)	1.51	(0.75–3.04)

* Data missing for one; HIV = human immunodeficiency virus; TB = tuberculosis; CI = confidence interval; aRR = adjusted relative risk.

**Table 4:** Treatment outcomes among tuberculosis patients started on treatment in Haiphong International General Hospital in Vietnam, from June to December 2018.

Treatment Outcomes	N	(%)
**Total**	**104**	**(100)**
Cured	58	(55.8)
Treatment completed	39	(37.5)
Died	2	(1.9)
Lost to follow-up	3	(2.9)
On treatment *	2	(1.9)

* Two patients are on second-line treatment and are likely to complete by April 2020.

**Table 5:** Delays in the TB care cascade among the patients attending the Haiphong International General Hospital in Vietnam, from June to December 2018.

Duration	Number Eligible	Number (%) with Valid Dates	Median Days	(IQR)
From visiting the HIGH to receiving the TB diagnosis test	494	487 (99)	0	(0–1)
From TB diagnosis to initiation of treatment	103	99 (93)	6	(1–17)

TB = Tuberculosis; IQR = Interquartile Range; HIGH = Haiphong International General Hospital.
